# Case series and literature review of multiple nodular sarcoidosis

**DOI:** 10.1186/1756-0500-6-394

**Published:** 2013-10-01

**Authors:** Hira Shahzad, Sameer Ur-Rehman, Kulsoom Fatima, Nadia Sharif, Ali Bin Sarwar Zubairi

**Affiliations:** 1 Medical College, Aga Khan University Hospital, Stadium Road, Karachi, Pakistan; 2 Department of Radiology, Aga Khan University, Stadium Road, Karachi, Pakistan; 3 Section of Pulmonary and Critical Care Medicine, Department of Medicine, Aga Khan University, Stadium Road, Karachi 74800, Pakistan

**Keywords:** Sarcoidosis, Nodular Sarcoidosis, Pulmonary Nodule(s)

## Abstract

**Background:**

Nodular lung disease is a rare presentation of sarcoidosis. Radiologically it can present as multiple pulmonary masses or solitary lung nodule.

**Case presentation:**

We report three cases of nodular sarcoidosis in young females of Asian origin who had initially presented with dry cough and worsening dyspnea non-responsive to initially administered antibiotics. Pulmonary nodules were discovered upon radiographic imaging in all three cases which raised concern for the possibility of neoplastic processes. Subsequent biopsies revealed granulomatous inflammation indicative of sarcoidosis. All cases responded very well to systemic corticosteroids.

**Conclusion:**

Sarcoidosis may present as nodular infiltrates which alerts the treating physician to other neoplastic and infectious diseases of the lungs. Appropriate workup may reveal the true nature of this disease and hence, simplify treatment.

## Background

Sarcoidosis is a multisystem granulomatous disease of unknown etiology, usually affecting young and middle-aged adults [1], commonly with pulmonary, dermatological and ophthalmological involvement. Lungs are the most commonly involved organ (almost 90%) with chronic irreversible changes leading to pulmonary fibrosis as evident in 20% of the cases [1]. In order to prevent chronic changes, early diagnosis and institution of early therapy becomes essential. However, given that Pulmonary Sarcoidosis is labelled as 'the great mimic’ in radiology [2], diagnosis becomes difficult on imaging alone owing to various forms of presentation and their simulation with metastatic or primary pulmonary malignancy or a myriad of infectious, inflammatory or vascular processes; especially when it presents with nodular mass(es). We report three such cases which initially presented with dyspnea along with several constitutional symptoms and on radiologic imaging were found to have nodules. On subsequent biopsies, these were diagnosed as rarely presenting nodular forms of sarcoidosis and were treated successfully with corticosteroids.

## Case presentation

### Case 1

**Figure 1:**
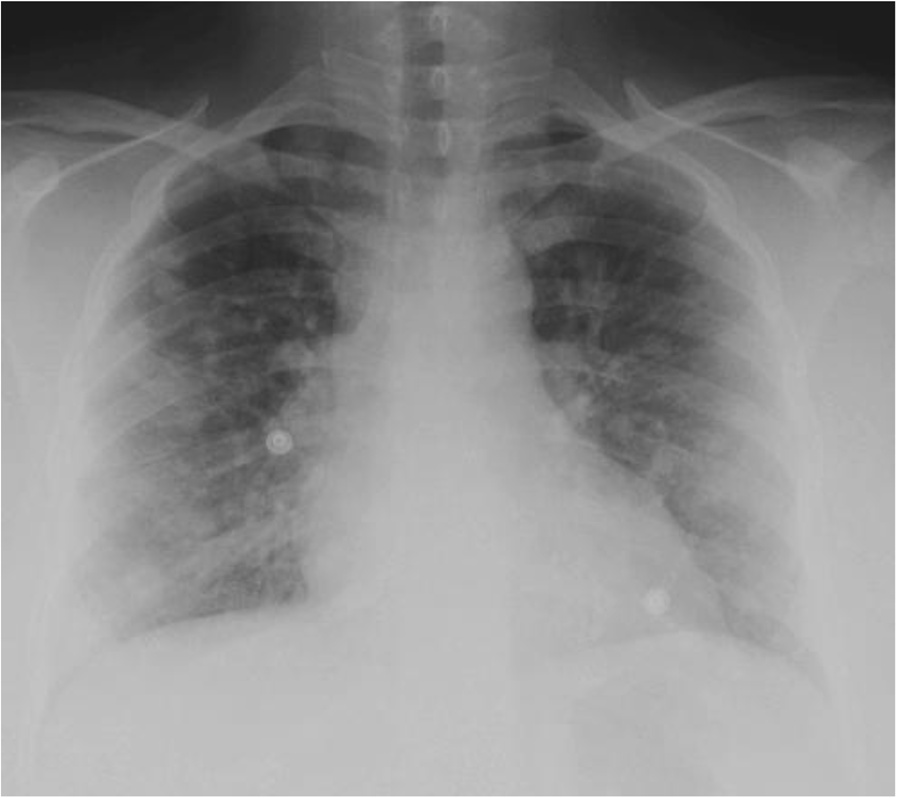
**Chest X-ray of Case 1: A 32 year- old female presented with a 2-month history of fever, dry cough, shortness of breath on exertion and weight loss.** She was treated with a number of antibiotics with no significant improvement. Chest radiograph showed extensive bilateral nodular infiltrates.

**Figure 2:**
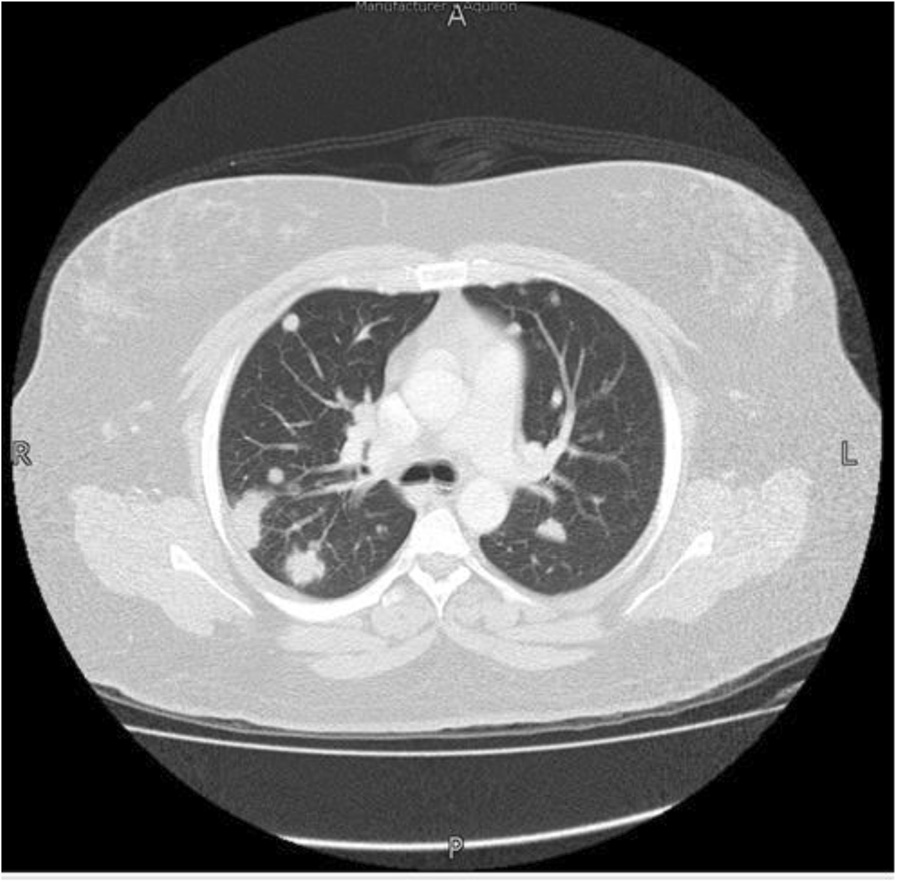
**CT chest of Case 1: This is the CT scan of the same 32 year- old female who had presented with a 2-month history of fever, dry cough, exertional dyspnea and weight loss.** She was treated with a number of antibiotics with no significant improvement. This scan further elaborated on the diffuse bilateral pulmonary nodular infiltrates which were evident on the chest radiograph shown in Figure 1. (A= anterior, L= left, P= posterior, R= right ).

A 32 year- old previously healthy non-smoker female, presented with a 2-month history of fever documented upto 103°F, dry cough, shortness of breath on exertion, arthralgias and weight loss of 2 Kg. She was treated with a number of antibiotics with no significant improvement. On physical examination she was tachypneic with a respiratory rate of 26/min and oxygen saturation at room air was 97%. She desaturated to 66% after a 6 minute walk test. She had pitting edema with tender red nodules over the shin consistent with eryrthema nodosum. Rest of the clinical examination was otherwise unremarkable. The complete blood count, renal function tests, liver function tests, C-reactive protein (CRP) and autoimmnune profile were within normal limits. The baseline serum angiotensin converting enzyme (ACE) level was 156 U/L. The transthoracic echocardiogram was normal. The chest X-ray showed bilateral extensive nodular infiltrates confirmed by high-resolution CT of the chest (Figures 1 and 2). She underwent video assisted thoracoscopic (VATS) lung biopsy which revealed non-caseating granulomatous inflammation and she was started on steroids. She showed dramatic clinical and radiological improvement within one month of initiation of corticosteroids.

### Case 2

A 43 year- old diabetic female, never smoker, presented with a 3-month history of low grade fever, dry cough and progressive shortness of breath. She had previously received multiple antibiotics and anti-histamines without any symptomatic improvement. Her physical examination was unremarkable. Laboratory tests including complete blood picture, urine D/R, CRP and autoimmune profile was within normal limits. Chest X-ray and HRCT showed mediastinal lymphadenopathy and predominant upper lobe nodular infiltrates. Video-assisted thoracoscopic surgical (VATS) biopsy of mediastinal lymph nodes and lung nodules showed chronic granulomatous inflammation with central necrosis. Tissue stains and cultures for microorganisms were negative. The possibility of necrotizing sarcoid granulomatosis was considered but there was no evidence of granulomatous vasculitis on lung pathology. The diagnosis of nodular sarcoidosis was made and she was started on steroids. The baseline serum ACE level obtained after histo-pathological confirmation was 70 U/L. After 2 months of treatment with systemic steroids, she showed significant clinical improvement and the serum ACE level came down to 15 U/L.

### Case 3

**Figure 3:**
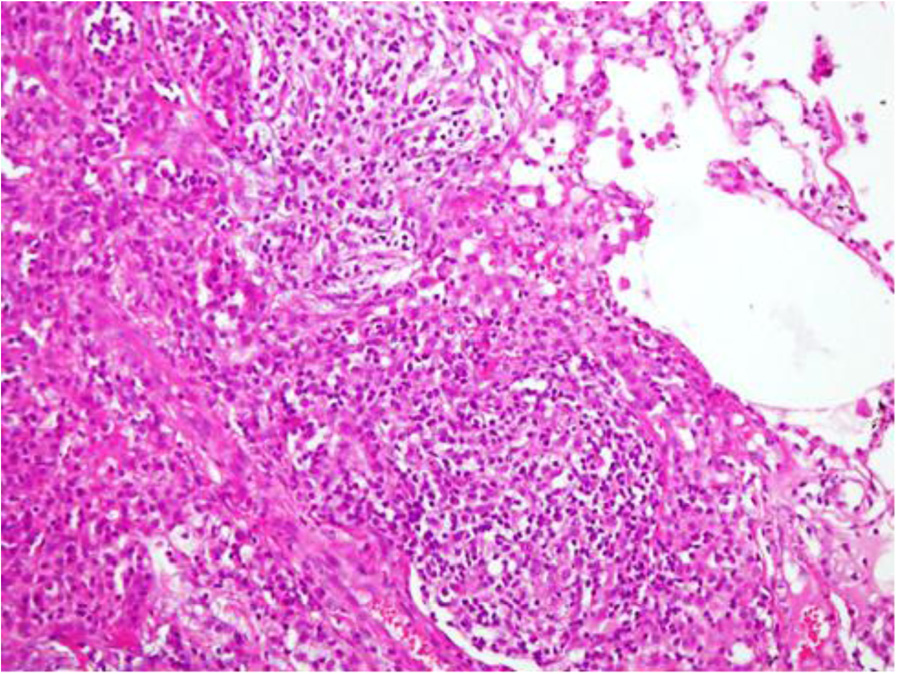
**Histopathology report from Case 3: A 29 year-old female who had presented with a history of cough and fever.** Chest X-ray and CT showed bilateral nodular infiltrates, for which she underwent CT-guided biopsy. Histopathology of the lung nodule showed non-caseating granuloma.

A 29 year-old female, non-smoker, who was diagnosed with Polycystic Ovarian Disease (PCOD) presented with a history of cough and fever for 25 days. She had delivered a baby 4 months back after hormonal treatment. On examination she was obese with significant hirsuitism, and had bilateral corneal congestion. Ophthalmologic evaluation did not reveal uveitis. The lab tests including complete blood picture, renal and liver functions and autoimmune profile were within normal limits. The chest X-ray and HRCT showed bilateral nodular infiltrates, for which she underwent CT-guided biopsy. Histopathology of the lung showed non-caseating granuloma (Figure 3). The serum ACE level was 110 U/L. She was started on steroids with remarkable clinical and radiological improvement on subsequent clinic visits.

## Discussion

Presentation of a nodular mass(es) on a chest radiograph is an ominous finding which alerts the physician to the possibility of a malignancy. As differentials, neoplastic lesions are enlisted as the more probable diagnosis rather than other benign differentials such as infections and granulomatous diseases like sacroidosis.

In sarcoidosis, pulmonary involvement occurs in up to 90% of the patients [1,2]. The usual radiological finding is of hilar and mediastinal lymphadenopathy with or without associated lung parenchymal involvement. The earliest published data on pulmonary nodular sarcoidosis was in 1952 by McCord and Hyman [3]. Nodular infiltration on radiography or computed tomography is a rare manifestation of the disease with an estimated prevalence of almost 2.4-4% as reported by Hansell and Sharma [4,5]. Since then, meagre data has been published on the nodular form of sarcoidosis [3,5-11]. Case series and other published data suggest that nodular sarcoidosis is more predominant in the younger age group (20– 40 years), African-American ethnicity and female gender [12]. This is in contrast with primary pulmonary neoplasms which tend to occur more commonly in males and in the older age group. Although sarcoidosis can present with constitutional symptoms, but these are not consistently reported in nodular sarcoidosis [7,10,12]. All of our patients had predominant constitutional symptoms of fever, fatigue and weight loss.

In sarcoidosis, functional impairment and radiological findings often do not correlate with each other [13,14]. Granulomatous inflammation can be demonstrated in either lung or lymph node tissue in nearly 100% of patients even in the absence of radiographic changes [15]. Interestingly nodular sarcoidosis is reported to be associated with minimal functional impairment [14], however two of our patients had significant complaint of breathlessness and one was associated with oxygen desaturation on 6 minute walk test, suggestive of diffuse interstitial involvement.

Nodules in sarcoidosis represent coalescing granulomas. They have the appearance of ill-defined opacities with irregular margins measuring 1**–**4 cm in diameter on CT. Sarcoid nodules typically emerge as bilateral opacities and are multiple in number frequently occupying the perihilar and peripheral lung zones [2]. 'Galaxy sign’ is obvious where small satellite nodules are seen to border the periphery of the larger nodules. This may be seen with other granulomatous and malignant lesions as well. Solitary lung mass or nodule is a rare radiologic finding of sarcoidosis. One study found multiple nodules in 82% of the cases whereas the remaining 18% had solitary nodules [12].

The radiological findings in nodular sarcoidosis are not specific and resemble other granulomatous and neoplastic diseases. Malignancy is a principal concern in these circumstances including both primary pulmonary neoplasms and metastatic lesions from genitourinary tumors, melanomas and sarcomas. Similarly mycobacterial and fungal infections may present with a similar radiological picture. Hence, tissue biopsy becomes a must to confirm the diagnosis. Findings in our patients were verified via histopathology which established the diagnosis of non-caseating granulomatous disease. We did not opt for bronchoalveolar lavage and transbronchial lung biopsy as our initial index of suspicion for sarcoidosis was low. Two of our patients underwent VATS biopsy and one had a CT-guided lung biopsy. A similar case was reported by Margaritopoulos et al. where radiological findings of a soft tissue mass at the hilum with bilateral diffuse lymphadenopathy in a 65 year old Caucasian female was highly suggestive of bronchogenic carcinoma which later turned out to be sarcoidosis [16]. In one of our patients, lung biopsy showed chronic granulomatous inflammation with central necrosis raising a possibility of necrotising sarcoid granulomatosis (NSG) although histopathology lacked vasculitis. This entity is a rare disease of unknown etiology which was first described by Liebow in 1973 [17]. The association of NSG with sarcoidosis is not yet established [18].

Patients with nodular sarcoidosis tend to have a favorable prognosis with significant improvement of the lung infiltrates [5,8,13,19]. Complete resolution of the masses, either spontaneously or with corticosteroid treatment, has been reported [5,7,8,20,21]. As our patients had significant constitutional symptoms and diffuse parenchymal lung disease they were treated with steroids. They responded very well showing complete clinical and radiological resolution of disease.

## Conclusion

Nodular sarcoidosis is an uncommon presentation of pulmonary sarcoidosis. It is found in young and otherwise healthy individuals with female predominance. Radiologically, it presents as nodular infiltrates mimicking other neoplastic and infectious diseases of the lungs. Appropriate workup may reveal the true nature of this disease and hence, simplify treatment.

## Consent

Written informed consent was obtained from each of the three patients for publication of this Case Series and any accompanying images. Copies of their written consent are available for review by the Editor-in-Chief of this journal.

## Abbreviations

ACE: Angiotensin converting enzyme; BHL: Bilateral hilar lymphadenopathy; Chest X-ray: Chest radiograph; HRCT: High resolution computed tomography; PCOD: Polycystic Ovarian Disease; VATS: Video assisted thoracoscopic surgery.

## Competing interests

The authors declare that they have no competing interests.

## Authors’ contributions

HS, SR, NS has collected analyzed and interpreted the patient data on nodular sarcoidosis under guidance of AZ. KF interpreted the radiologic imaging. HS, SR and AZ were major contributors in writing the manuscript. There was no source(s) of funding for any author and for the manuscript preparation. All authors read and approved the final manuscript.
